# Factors contributing to the quality of nursing care in Gauteng province hospitals

**DOI:** 10.4102/curationis.v48i1.2653

**Published:** 2025-03-25

**Authors:** Nomali S. Sabelo, Sibusiso M. Zuma

**Affiliations:** 1 Department of Health Studies, School of Social Sciences, University of South Africa, Pretoria, South Africa

**Keywords:** holistic care, hospitals, nursing care, professional nurses, quality, work environment, workload

## Abstract

**Background:**

The South African public is not satisfied with the level of healthcare rendered by South African health institutions, particularly in Gauteng province; this is evidenced by the concerns raised by the South African Health Ombudsman, who, as of 2021, received more than 2000 complaints from the public, of which 40% were from Gauteng province. This is supported by South African Nursing Council (SANC)’s unprofessional report, which reflected the increase in poor nursing care cases.

**Objectives:**

The objectives of the study were to identify the factors contributing to the quality of nursing care at the provincial hospitals of Gauteng province and to make recommendations for improving the quality of nursing care in hospitals.

**Method:**

The study utilised an exploratory descriptive qualitative design. The data were analysed following Colaizzi’s steps of data analysis, during which 4 themes and 9 sub-themes emerged. The study population consisted of registered professional nurses working in the two hospitals. The sample consisted of 12 registered professional nurses.

**Results:**

The study identified three positive factors, namely, nursing as a calling, supervision rounds and staff appreciation. Six negative factors that contribute to the quality of nursing care were identified as the lack of human and material resources, administrative challenges, unsupportive management, nurse-related factors, attitudes of patients, and private versus public health institutions.

**Conclusion:**

The study revealed the positive factors that promote the quality of nursing care. The negative factors affecting the quality of nursing care were found to be more than the positive factors.

**Contribution:**

The study presented factors affecting the quality of nursing care and makes recommendations that can be utilised as basis for improving the quality of nursing care. The study findings and recommendations can be used to develop programmes to support nurses to provide dignified and quality patient care in the hospital.

## Introduction

Quality nursing care has an impact on patient outcomes and health facilities’ reputations. The World Health Organization (WHO) reports that between 5.7 and 8.4 million deaths are associated with poor quality of care in low- and middle-income countries, yearly (WHO 2020). In South Africa, the quality of nursing care is an important factor in the provision of holistic patient care. Maphumulo and Bhengu (2019) report that the South African Government has put in place several quality improvement strategies to better the provision of healthcare to the public and yet the quality of healthcare is still reported negatively by the public. Poor quality of nursing has negative effects on the health system, including increased litigations related to unethical behaviour such as poor nurse–patient interactions, negative attitudes and poor documentation, which could be avoided with proper nursing care (Mathibe-Neke 2020).

In South Africa, the provision of nursing care is guided by laws such as the *Nursing Act*. According to the *Nursing Act* (Act No.33 of 2005), one of the nurses’ important functions, as stipulated by the South African Nursing Council (SANC) under the scope of practice for nurses, is the provision of quality nursing care (South Africa 2005).

### Problem statement

The SANC statistics of unprofessional conduct indicate that there is an increase in the reported cases of unprofessional conduct in nursing in the Gauteng Province (Government Notice 2020). Furthermore, the element of quality nursing care and patient advocacy seems not to be considered in nursing nowadays, which is evidenced by the incident that occurred in 2019 in Mamelodi Hospital whereby a 76-year-old female was found tied to a steel bench by her hands and lay on the floor for a day and night after a family member reported that she had left the patient the previous day (South African Human Rights Commission [SAHRC] 2019). Even though the use of physical restraint prevents harm and injury to confused patients, its use without constant supervision by the registered nurse to satisfy the patient’s needs poses a threat to the patient’s care (Palese et al. 2020:929). It has been found that treating patients with respect and dignity following ethical principles is therapeutic and leads to safe patient care and patient satisfaction (IIkafah, Tyas & Haryanto 2021; Naidoo & Van Wyk 2019).

Bloom (2021a) reports that in Gauteng public hospitals, adverse events cases, including poor quality of nursing care, increased from 4170 in 2019 to 4701 in 2020, with Dr. George Mukhari Hospital being the highest with 1022 cases. Researchers have identified that patients’ illnesses may be worsened by adverse events from health institutions that could have been prevented, which may result in prolonged hospitalisation, physical harm, or even death and high costs for the government (Bloom 2021b; Kjellberg et al. 2017:17; Tessier et al. 2019:E879). Based on the increasing reports about poor quality nursing care in Gauteng hospitals, the researcher identified a need to investigate the factors contributing to the quality of nursing care at the two provincial hospitals of Gauteng province and to make recommendations for improving the quality of nursing care in hospitals.

### Purpose of the study

The purpose of the study was to explore and describe the factors contributing to the quality of nursing care at the provincial hospitals of Gauteng province and to make recommendations for improving the quality of nursing care in hospitals.

## Research methods and design

Qualitative exploratory descriptive design was used for the study. The design was suitable for the study as the focus was on narrative content; four main themes emerged during the data analysis process.

### Setting

The study setting was two public hospitals in the Tshwane Health District, Gauteng, South Africa.

### Study population and sampling strategy

The study’s target population was all registered professional nurses working in the two hospitals. Purposive sampling was utilised to select registered professional nurses. Inclusion criteria were female and male registered professional nurses with 10 years or more of working experience. Exclusion criteria were registered professional nurses (PN) with less than 10 years of working experience, enrolled nurses and auxiliary nurses.

### Data collection

Data were collected at two public hospitals using a semi-structured individual interview guide. The interviews were conducted between November 2022 and January 2023. One central question asked was, ‘What are the factors influencing the quality of nursing care in your hospital?’ Participants’ permission was requested to audio-record the interviews, which lasted no more than 60 min per session, except for two interviews – one extending to 1 h and 20 min and the other to 1 h and 15 min.

Data were collected until saturation was reached at the 12th interview, where no new information was obtained.

### Data analysis

The tape-recorded verbatim data were transcribed into written form, and significant statements were identified which were then interpreted into themes. The study adopted Colaizzi’s seven procedural steps for analysis (Polit & Beck 2017). An independent expert in qualitative data analysis assisted the researcher in co-coding, she received audio-taped interviews and transcripts for analysis. The researcher and the co-coder had a consensus discussion about the themes and sub-themes of factors affecting the quality of nursing care.

### Trustworthiness

The researcher ensured the trustworthiness of the research process by applying credibility, dependability, confirmability, and transferability criteria, to ensure that the overall outcomes of the study are reflecting the participants’ true explanation and version, and not the preferred viewpoints of the researcher (Polit & Beck 2017). Face-to-face interviews with the participants lasted a long time whereby the researcher built rapport and trust to enable the free flow of in-depth information, the participants’ non-verbal cues were noted, and probing questions were posed to get a clear understanding of the factors contributing to the quality of nursing care (Brink, Van der Walt & Van Rensburg 2018). Triangulation was achieved by getting information sources from two different hospitals as well as by tape recording the interviews and taking field notes at the same time (Brink et al. 2018). Peer debriefing was achieved by constantly requesting assistance from colleagues who have used a similar method to verify the correctness of the process followed. The researcher maintained the use of similar tools for collecting data in the two hospitals and kept the records of the study proceedings to ensure dependability. Confirmability was ensured by using an audio recorder to capture participants’ responses in their original and unchanged state, which were transcribed verbatim and analysed to determine themes and associated sub-themes and then submitted to the co-coder for an independent verification and relevance check (Polit & Beck 2017). Transferability was ensured through a detailed description of the factors contributing to the quality of nursing care. Participants were purposefully selected based on their location or setting and years of work experience; data were collected from the participants who met the inclusion criteria, until data saturation was achieved (Brink et al. 2018; Leavy 2017; Polit & Beck 2017).

### Ethical considerations

Ethical clearance to conduct this study was obtained from the University of South Africa, College of Human Sciences Research Ethics Review Committee (No. 30863015_CREC-CHS-2022) followed by permission from the Gauteng Provincial Department of Health, the District Executive Officer of Tshwane District, the Chief Executive Officer, and the Clinical Manager of the two hospitals where the study was conducted. The researcher observed the principles of justice and beneficence, as well as respecting human dignity throughout the study. Participants were not exposed to any form of physical, emotional or psychological harm (Polit & Beck 2017); all interview questions were not disturbing to participants’ emotional and psychological aspects. The participants’ right to protection from exploitation was respected throughout; the participant information sheet and informed consent form were written in simple English; all participants were notified of their right to withdraw from the study if they felt they could not cope with the interviews; and the time to spend was communicated to the participant upfront for the participant to decide to partake in the study or not (Polit & Beck 2017). The participants were treated with dignity and respect; the interview time was set by individual participants depending on availability; and they were thanked for partaking in the study (Gray, Grove & Sutherland 2017). The researcher fairly applied the sampling inclusion and exclusion criteria to ensure that no participant was discriminated against (Brink et al. 2018). The information leaflet and informed consent form ensured participants’ awareness of their legal right of choice; the e-mail address, phone numbers, and the name of the researcher’s academic supervisor were provided to participants to be able to report any unwarranted conduct on the part of the researcher. The right to privacy was ensured by keeping the anonymity of the participants; their identities do not appear anywhere in the study; on analysis, the participants were allocated numbers for identification; and field notes were taken with their permission. The field notes and audiotaped data were only accessed by the researcher, the supervisor and the co-coder (Brink et al. 2018).

## Results

### Demographic profile of the participants

The interviewed participants’ age ranged from 35 years to 64 years. Out of the 12 participants, 11 were females and 1 was male. All participants were registered with the SANC. Seven participants held their highest qualifications in various post-basic diplomas: three in Paediatric Nursing Science, two in Primary Health Care Nursing Science, one in Critical Care Nursing Science, Nursing Education, and Administration, and one in Orthopaedic Nursing Science, Nursing Education, and Administration. All registered nurses had more than 10 years of work experience.

### Presentation of findings

The findings are discussed under four emerging themes:

1. Concept definition of quality in nursing care.
2. Positive factors affecting the quality of nursing care.
3. Negative factors affecting the quality of nursing care.
4. Recommendations for improving the quality of nursing care.

### Theme 1: Concept definition of quality in nursing care

The participants gave various answers, but all suggested that quality nursing care is comprehensive nursing care – nursing care that focuses on all aspects of the patient’s life in totality:

‘It is total nursing care of a patient, nursing a patient in totality, yah, I must see to the needs of the patient.’ (PN1, Female, 55 years old)

‘You nurse the patient holistically, *neh*, you nurse the patient physically, spiritually, socially, emotionally, and psychologically, you meet all the aspects of the patient *neh*, you don’t only concentrate on what brought the patient to the hospital.’ (PN2, Female, 46 years old)

‘[*By quality nursing care*] we mean comprehensive nursing care.’ (PN12, Female, 46 years old)

Nursing care must be rendered by adopting a holistic approach because, in most cases, the patient may not be aware of other problems they may have and only report the presenting signs and symptoms, meanwhile, other problems may also contribute to the problems they are presenting. It needs a nurse to be able to assess the patient in all spheres – physical, psychological, social and spiritual – and intervene accordingly to meet the patient’s needs. The study findings are in line with Lateef and Mhlongo (2020) who advocate for patient-centred care, which is developed to enhance holistic patient care as it promotes the focus on the patient’s needs and values, thus encouraging dignity and respect for the patient.

### Theme 2: Positive factors affecting the quality of nursing care

#### Sub-theme 2.1: Nursing as a calling

Some participants’ statements show that most nurses are nurses by calling. They can identify the weaknesses in the profession and the factors that bring its name into disrepute, and they become exemplary by going the extra mile in their job and implementing a range of initiatives:

‘I am working with diabetics, *neh*, so I have this bond with them, *neh*, like this one [*she played a voice message for a patient who was reporting her problem that caused her to miss her clinic appointment, it appeared that the patient is having depression as she said she feels like keeping to herself and close herself in the house and at times she feels like she can buy poison and poison herself and the children*] So this is what we are doing *neh*, So I told her to come and then I will send her to the psychologist.’ (PN2, Female, 46 years old)

[*I*]n this ward we have a book for complains and complements, you know, which in the morning we do rounds and ask the patients how they are, because I told them no one must leave the patients with the complaints that were not attended to and resolved, you see. And we tell the patients that they must talk if they are not feeling happy about anything.’ (PN3, Female, 49 years old)

The above statements reflect that nursing as a calling produces close nurse–patient relationships, enabling the nurse to diagnose all the patients’ problems and healthcare needs. It breaks the barrier and causes the patient to talk freely about their health problems in a relaxed state and the nurse will be able to observe the patient’s emotions and untold concerns and thus perform relevant interventions such as referring the patient appropriately.

#### Sub-theme 2.2: Supervision rounds promote quality of nursing care

The supervision rounds of the wards were identified as a step in the right direction of quality nursing care because staff members work towards achieving quality nursing care:

‘And I think another positive, since they have introduced this thing of assessment of supervision, our managers visit different wards and they give us feedback and we implement whatever that is positive to improve patient care … they benchmark with other wards, we adopt what is done positively in other wards; and whatever that is negative the quality improvement plans are done.’ (PN6, Female, 38 years old)

‘[*O*]ur CEO is not office bound he moves around … the Nursing Service Manager work with the wards to identify problems. Just to motivate people to work hard …’ (PN10, Female, 55 years old)

#### Sub-theme 2.3: Staff appreciation

Staff appreciation goes a long way in promoting quality nursing care. Staff satisfaction plays a role in providing quality nursing care, as it promotes staff morale and encourages transcendence that results in patient satisfaction:

‘[*M*]anagement has organised a motivational speaker through the Chaplain, we received some cups on the nurses’ day at least that peps up the morale of the staff. And when requesting for overtime they do not hesitate they give you and the money for overtime is paid separately from the salary, there is also EWP if you not feeling well you go to EWP it helps, and our Chaplain is doing a very good job if she can here you just get healed.’ (PN5, Female, 35 years old)

### Theme 3: Negative factors affecting the quality of nursing care

The interviews revealed that negative factors affecting the quality of nursing care are lack of human and material resources, administrative challenges, unsupportive management, nurse-related factors, attitudes of patients, and private versus public health institutions.

#### Sub-theme 3.1: Excessive workload

The interviews reflected that the shortage of staff is affecting the quality of nursing care, especially in public institutions in South Africa; the nurses become overwhelmed by the work overload caused by staff shortages, which is worsened by a shortage of material resources and the expectations to provide quality nursing care despite being faced with such shortages:

‘If people are overworked there is lack of concentration, like myself now I have not eaten for the whole day now how can you work effectively when you are tired? So, concentration sometimes, mistakes can happen, so (shaking shoulders) really, when you are exhausted you cannot function well, sometimes you find that at 3 o’clock is the first time that the nurse go for tea, you see the staff here in ICU they didn’t go for tea, because of shortage of staff, so how can one concentrate when the brain does not have sugar.’ (PN5, Female, 35 years old)

‘[*Y*]ou find that at 12 the resuscitation trolley is not checked, and you will be found at fault that you did not check before start working, but you were busy the whole time since arrival, you have not even gone for a tea break.’ (PN7, Female, 37 years old)

#### Sub-theme 3.2: Lack of material resources

Participants’ statements show that lack of resources is a major hindrance in the provision of quality nursing care especially in the South African public health facilities and it unfairly leads to nurses being viewed as incompetent by the public, yet it is beyond their control:

‘[*S*]hortage of consumables, like NG tubes, giving sets, feeding tubes …, because for malnourished children we have to insert NGT, infusion sets, the stock they give us is not enough, sometimes you find that we do not have linen savers it is out of stock we have to go and borrow from other wards in that staff shortage.’ (PN11, Female, 57 years old)

‘So if you are going to give medication and IVIs [*intravenous injections*] alone somebody is going to suffer, there is shortage of supplies e.g. syringes you have to go around to other wards borrowing, the time is going, you go to the stores you find people sitting.’ (PN1, Female, 55 years old)

#### Sub-theme 3.3: Administrative challenges

Administrative challenges were also reported to hinder the provisioning of quality patient care and poor communication among the staff members regarding patient care, which has a negative impact on the quality of nursing, starting from the security at the hospital entrance to the administrative clerks. Excessive documentation was also identified to be taking nurses’ time away from patient care:

‘There is a barrier between administration and nursing there is no proper communication, like recently they have changed the registration number system we were never involved in that, we were not told how are the new numbers going to work, they provide two numbers we don’t know which is the important number to use, they just changed the system which has delays the patients in the morning because they must change the registration numbers to the new system, and they don’t change all the patients and they delaying us.’ (PN8, Female, 55 years old)

‘They [*nurses*] focus on the documents more than to patient and the patient feel lost, so communication with the patient is less, at the end of the day patient feel that is not well cared for, lots of recording to be done is also a contributing factor to not having enough time to talk to the patient, because before admitting the patient there are lot and lot of records to be done, and then there will be questions more on the records than the patient, as long as the records are fine everything is fine.’ (PN4, Female, 46 years old)

‘Sometimes we become confused and frustrated because a lot of documents affect patient care they don’t come to support us.’ (PN6, Female, 38 years old)

The participants’ responses reflect that the nursing profession is dealing extensively with records for patients and clients, and the time for patient care is reduced.

#### Sub-theme 3.4: Unsupportive management negatively affects the quality of nursing care

The participants revealed that the management adds to the staff’s frustration by not providing support. At times the registered nurses are forced to work outside their scope of practice; furthermore, the management does not treat staff fairly:

‘[*A*] big problem, you know managers have a problem not in this hospital alone, managers do not act immediately when problems are reported, but when something wrong happen they come quickly because they know they are also involved.’ (PN12, Female, 46 years old)

‘You know if you working hard, we are working hard here, you need someone who can say we care, just say we care, nothing else, just somebody who can say, not somebody to come and look for the problems in the ward, we need people who are coming here with positive attitude, then you become to have positive attitude, and if you come with a positive attitude I also have positive attitude, those things affects the workers negatively … if we can have a person who can come and say hi I was just checking on you, you are working hard and thank you for that, just that.’ (PN1, Female, 55 years old)

#### Sub-theme 3.5: Working beyond the scope of practice

The participants verbalised that sometimes they are delegated duties that are beyond their scope of practice, which puts the patients’ lives in danger and exposes those nurses to the risk of being removed from the nurses’ roll by the controlling body. When concerns are raised regarding these unsafe assignments, they are labelled as people who do not want to work:

‘[*M*]*ina* I am a paediatric specialist nurse, I am able to nurse very ill patients but with oxygen prongs, not ventilated patients because it’s not critical care there are no ventilators in this ward you can see from entrance to the end because it’s not critical care unit. So, they want to put me in danger as well as patients because I don’t have that skill.’ (PN12, Female, 46 years old)

‘Affecting negatively, for example I was taken to do two weeks crash course, and with that little knowledge I was expected to nurse a ventilated patient, you understand, now what good I am going to do to the patient.’ (PN7, Female, 37 years old)

‘It is not that we do not want to work, no, we want to work but in a proper way, that will not harm the patient and that will not harm our qualifications as well, not to be subjected to this oppression, because if something happens, I will be alone to answer to Council, they don’t even come to support.’ (PN11, Female, 55 years old)

#### Sub-theme 3.6: Favouritism, unfair treatment and abusive management

Participants verbalised that they sometimes receive unequal treatment by the management, which reflects favouritism in issues including unfair discipline and the process of promotion to senior positions, where promotions are based on favoritism rather than following correct procedures:

‘And then coming to our managers eih, they have become laws to themselves, we have very bad … and this has never happen before, ‘usilomang’, the whole manager asking who are you, it’s very nasty its nasty yah, mina ngingena ngihlale la, on arrival I sign the register and come and seat here, I go out and take patients files and come back to seat here and consult the patients, after finishing the patients I take my lunch box and eat here, uyabona manje I isolation, because you are not happy … they give people the posts by favouritism, you don’t get the post because you have an insight for that department.’ (PN9, Male, 55 years)

‘Sometimes I feel that we don’t have a voice here whereas in the private when you come with a suggestion the whole ward get interested to know more and you explain in details, were even having a form we used identify high risk, we identify and deal with it and identify another one same thing happen.’ (PN7, Female, 35 years old)

‘One day one matron told me something hurting, and I am still hurt it’s just that … she told me I must lie sometimes; I told her I cannot it’s just me, she said “there is no monument that will be built for me,” what does that mean?’ (PN1, Female, 55 years old)

Abusive management is a reported problem in South Africa, especially in public health facilities as evidenced by research conducted across three provinces, Western Cape, Free State and Gauteng as subordinates display a lack of confidence and lack of passion for their jobs (Johnson 2023:1).

### Theme 4: Recommendations for improving the quality of nursing care

Continuous professional development was recommended for the staff to render quality nursing care and achieve patient satisfaction:

‘I think continuous in-service on new developments in nursing and patient care, can aid in rendering quality nursing care, and staff satisfaction surveys can also help to identify staff concerns and intervene.’ (PN6, Female, 38 years old)

‘[*Y*]es, staff development can help to uplift the standard of nursing care, it can help a lot, if they do not develop us who is going to update us with the new things, you know nursing care changes every time we need to be updated.’ (PN12, Female, 46 years old)

Participants recommended that professional conduct should be encouraged to maintain the dignity of the profession:

‘[*M*]*ina* what I can recommend now, let’s dwell on the values and ethics of nursing, lets revive them … everywhere, it can start at the colleges because we are dealing with a person we do not know their upbringing, so that even if the person has come to nursing by chance but, he get to be taught the values and ethics so that get to tune.’ (PN9, Male, 55 years old)

‘[*N*]urses if they can revive their scope of practice and their pledge and not just sing it, they need to take it to heart and understand what it say what it means. And the I care values are important we need to reinforce them, maybe they will change … things are getting worse every day.’ (PN10, Female, 55 years old)

Participants recommended that more staff be employed for the correct or satisfactory nurse–patient ratios for appropriate workloads to deliver quality nursing care; furthermore, strategies to retain staff should be adopted, such as creating a positive work environment with adequate working resources, satisfactory salaries, supportive management, and recognition for job well done (Arif et al. 2020:231):

‘I think if they thank people like our CEO every year there is CEO awards, whereby they chose people that are outstanding in their performance and they reward them, just to appreciate and encourage them [*uhm that is good*] I think that can contribute positive … being thankful and recognize make you to work harder.’ (PN10, Female, 55 years old)

‘I think rotation can work because people stay in one unit for years, but I don’t know because other people work well in the units they know, maybe if they can take those KKMs to work somewhere else. Some do not know the work, so they hide themselves and have attitudes so that they are not seen that they do not know.’ (PN7, Female, 35 years old)

Some of the participants suggested that the clinical facilitators must be more visible and teach practical in the clinical facilities rather than theory to ensure the competence and confidence of students on completion of training. Some suggested that the nursing training be offered in public nursing colleges because products from the Universities and private colleges show lower standards of performance:

‘[*S*]o I think you need to revisit the curriculum and to put more practical, because it no longer done like before. You cannot teach something that you not passionate about nursing is practical.’ (PN9, Male, 55 years old)

‘They need to bring nursing education to hospitals, and stop at the universities, the universities aih, and they are very arrogant shame. The private, private do not care about the character of a nurse, they only care about money, eih I don’t know.’ (PN10, Female, 55 years old)

## Discussion of findings

Multiple factors positively and negatively affecting the quality of nursing care were identified. The findings showed that nurses have the same understanding of what is entailed by the term quality of nursing care. Some explained it as comprehensive, while others explaining it as total nursing care, all of which talks to the holistic. The findings are in line with Lateef and Mhlongo (2020:23) who advocate for holistic patient care that is physical, psychological and spiritual care, as it promotes the focus on the patient’s needs and values, thus encouraging dignity and respect for the patient.

The study revealed that there are factors that positively affect the quality of nursing by nurses and management initiatives to improve the quality of nursing care, which include encouragement of co-workers to treat patients and their families with dignity and respect. They also encourage patients to write their complaints and compliments and they redress shortcomings. The participants stated that they carry out monthly audits in the units and generate quality improvement plans to ensure the quality of nursing care.

It was stated also that the management implements various strategies to improve the quality of nursing such as performing monthly assessments of all units in the hospital to ensure the achievement of the ideal hospital; this was identified to be improving the quality of nursing care, as the employees work towards achieving high scores for their units. The recognition and reward of the best employee by the management was considered to contribute to job satisfaction and encourage other staff members to work with dedication to also be rewarded. Washeya and Fürst (2021:1) attest that staff recognition of good work promotes staff satisfaction, working with dedication and retention. Correspondingly, Mathibe-Neke (2020:52) states that dissatisfaction among nurses leads to low morale and low standard of care, which constitutes malpractice and results in litigations.

Negative factors like a lack of resources emerged as a factor that affects the quality of nursing care; this was mentioned by participants when stating that they experience work overload because of inappropriate nurse–patient ratios resulting from a shortage of staff. The lack of material resources was also found to affect the quality of nursing care when the participants stated that they must borrow working materials from other wards before they start to provide nursing care, which causes delays to patient care and dissatisfaction as well as nurse exhaustion. Morton et al. (2020:1024) indicate that lack of resources is a major hindrance in the provision of quality nursing care, especially in South African public health facilities, and it unfairly leads to nurses being viewed as incompetent by the public, yet it is beyond their control. Similarly, Mmadi and Sithole (2019:1) report that in some instances nurses must use their cell phones and torches to deliver babies because of the absence of suitable resources. Mogakwe, Ally and Magobe (2020:1) found that a lack of resources like medical supplies, stationery, and equipment and non-maintenance of available equipment cause non-compliance with quality standards and affect the quality of nursing care. The participants mentioned that one of the major causes of lack of resources is budget cuts. This can be interpreted as denied access to healthcare, whereas inability to receive proper treatment could lead to morbidity and death; this is a violation of the right to life and dignity, which are the constitutional non-derogable rights in South Africa (Dhai & Mahomed 2018:8). The research showed that in addition to work overload, nurses perform non-nursing duties; this was clear when participants stated that they must fetch supplies from stores, medication from the pharmacy, and linen from the linen room when it is not delivered. Weston (2022:152) confirms that most of the nurses’ time is spent on non-nursing duties because of staff shortages and non-commitment by other support staff members.

Administrative challenges were also found to be another factor that affects the quality of nursing care, which became obvious when the participants verbalised that there is poor communication between nurses and administrative staff regarding patients’ files and changes in the filing system, leading to loss of important patient history that disturbs the patient’s continuity of care and treatment, and poses a risk to life. Loss or fragmentation of records causes delays in the diagnosis and management of patient problems (Choi et al. 2021:296). Proper and effective communication among the healthcare team, patients and families is crucial in ensuring quality patient care and safety, and thus the healthcare team needs to understand that their jobs in different sections or departments are interdependent and all aim to reach the organisational common goal, which is to provide quality patient care (Kelly et al. 2020:1). The findings showed that excessive documentation that is required takes time away from patient care. The participants mentioned that they spend less time with patients, to allow time for documentation. Some participants stated that additional documentation is frequently introduced for quality assurance which further takes time for patient care as they have to under go orientation on new documentation requirements. Research confirms that excessive documentation and regulatory demands take nurses’ time away from patient care and lead to missed patient care and negative patient outcomes; on the other hand, it causes documentation burden and burnout to nurses (Hobensack et al. 2022:439).

The participants verbalised that there is a lack of management support, a crucial factor that negatively contributes to the quality of nursing care. Some stated that they are delegated assignments or duties beyond their scope of practice. This poses a risk to patients’ lives and their jobs because each nurse category has a scope of practice as per the SANC prescripts. The management was said to be treating staff unfairly where there is unequal punishment for similar problems because of favouritism; some are also given positions based on favouritism. Washeya and Furst (2021:1) report that a negative working environment, including favouritism and lack of transparency, causes nurses to leave their positions for alternate employment, which leads to more shortages.

The study found that some nurse-related factors also affect the quality of nursing care, such as nurses’ well-being. Participants stated that a negative working environment affects the physical and psychological health of nurses and leads to burnout, which harms the quality of nursing care. Unsatisfactory salaries were considered to contribute to job dissatisfaction; some participants reported that because of poor working conditions and lack of professional development, they no longer have passion for their jobs. Some suggested that SANC’s continuous professional development strategy implementation be hastened so that employers will have no choice but to give staff members time for professional development equally because it will be a requirement for all nurses to renew their practice license. Congruently, Ford and Thareja (2023:10) state that nurses leave the profession because of staff shortages, unsatisfactory salaries and not being appreciated. Nurses become motivated when they are paid well for their services (Munger 2019:16). Maintenance of excellent nursing demands continuous professional development requiring identification of staff development needs, if the staff is not developed professionally and not satisfied with the job, they underperform or do a substandard job and they display a lack of passion for the job (Bassiouny & Elhadidy 2022:402).

The participants stated that some nurses do not maintain professionalism in that they do not wear the proper uniform for a good professional image, and some exclude patients in decision-making about their care by using language not understood by patients, which may lead to non-compliance to treatment because of a lack of information. This reflects disrespect for patients and a lack of ethics and professional practice. Respect and professional appearance play a major role in the dignity of the profession, and how nurses wear their uniform and communicate to patients brings hope to clients and patients; it boosts the self-confidence of a nurse and encourages perfection in nurse’s functioning, resulting in the provision of high-quality nursing care (Cao et al. 2023:22; Ndirangu et al. 2021:20).

The study found that the newly qualified nurses show deficiencies and insufficiencies in their professional roles, which was revealed when the participants stated that new nurses lack knowledge and skills in nursing; they do not carry out procedures correctly, decide about patient care even if delegated and do not show commitment to their work. Some participants stated that the product of some private nursing schools is incompetent, while others stated that nurses trained in universities lack professional competency and confidence in practice; they know theory only, which does not help because nursing needs integration of theory to practice.

The nursing education and training were perceived to be ineffective by the participants who stated that the clinical accompaniment of students is not done properly. Clinical facilitators do not visit regularly for accompaniment, and when they do, they call all the students to offices or aside to teach theory, which leads to a lack of skills and the production of incompetent nurses. This is confirmed by Mabusela and Ramukumba (2021:1) who reported that newly qualified nurses are not work-ready and not skilled enough to practice independently; this is because of inadequate supervision during training where students are made to cover the shortage of staff. Similarly, Rabie, Rabie and Dinkelmann (2020:19) report that newly graduated registered nurses experience various challenges on entering the practice environment, including transitioning from being students to being professionals, overwhelming workloads, poor organisation of patient care, lack of knowledge, and insufficient failure to follow protocols; some show lack of independence, confidence and deficiencies in their professional roles, which is because of insufficient practical experience and lack of clinical accompaniment during training.

The patients’ attitude was also identified as negatively contributing to nursing care. Participants reported that some patients arrive at hospitals with preconceived notions and become rude to nurses, while others expect to be favoured over others. Some participants mentioned that some patients delay seeking medical help; they start with self-treatment or traditional methods and only come to the hospital when it is too late. These participants then expect wonders and blame the hospital if they are not healed (Serpa et al. 2020:79).

Some participants stated that the quality of nursing care rendered in private institutions is better than in public institutions; there are adequate resources and proper infrastructure in private institutions and the opposite applies to public institutions. This is supported by Mmadi and Sithole (2019:1) by report that private health facilities are better preferred by the public because they are well-resourced compared to public health facilities, which are challenged by overcrowding, shortage of human and material resources, and staff attitudes.

Participants’ recommendations to improve the quality of nursing care included providing equal opportunities for continuous professional development for all staff members.

Participants advised employment of adequate and suitable staff to maintain acceptable nurse–patient ratios and that they should be provided with adequate material resources, teaching support staff about the importance of their contribution to quality nursing care, to save nurses from having to carry out non-nursing duties.

Participants advised that clinical accompaniment should be done vigorously to ensure competent and confident nurses for the safety of patients and quality nursing care. Others advised that nursing training should be offered in public colleges only. Some even mentioned that going back to basics is the solution for quality nursing care.

### Limitations

The limitations of this study were that it was conducted in the Gauteng province in two hospitals only. As a result, the findings may not be generalised to all other hospitals in the province or to other provinces. Another limitation is that the study focused only on registered nurses, which disadvantaged other experienced categories of nurses who could have contributed to the study.

### Recommendations

#### Nursing practice

Nurse managers should create a positive work environment where there are adequate resources coupled with continuous professional development that contributes to professional nursing practice.

#### Nursing education

Nursing education institutions, health institutions, and Provincial Health Departments should collaborate in facilitating clinical training of students.

New graduates into the profession should be allocated a mentor to promote competency, caring and patients safety.

#### Nursing research

Further research should be conducted into the factors contributing to the quality of nursing care.

Development of frameworks and guidelines for improvement in the quality of nursing care.

## Conclusion

The study reveals that there is a need for collaboration among various levels of management in nursing practice and nursing education to harmonise the working environment and create a positive work environment that fosters patient and nurses’ satisfaction.
